# PERCUTANEOUS RADIOFREQUENCY ASSISTED LIVER PARTITION WITH PORTAL VEIN EMBOLIZATION FOR STAGED HEPATECTOMY (PRALPPS)

**DOI:** 10.1590/0102-672020180001e1346

**Published:** 2018-03-01

**Authors:** Mariano E. GIMÉNEZ, Eduardo J. HOUGHTON, C. Federico DAVRIEUX, Edgardo SERRA, Patrick PESSAUX, Mariano PALERMO, Pablo A. ACQUAFRESCA, Caetano FINGER, Bernard DALLEMAGNE, Jacques MARESCAUX

**Affiliations:** 1 University of Buenos Aires, Buenos Aires, Argentina; 2 Docencia Asistencia Investigación en Cirugía Invasiva Mínima (DAICIM) Foundation, Buenos Aires, Argentina; 3 Institut Hospitalo-Universitaire (IHU), Strasbourg, France; 4 Institut de Recherche contre les Cancers de l´Appareil Digestif (IRCAD), Strasbourg, France; 5 Novel Hôpital Civil, University of Strasbourg, Strasbourg, France; 6 Hospital Bernardino Rivadavia, Buenos Aires, Argentina; 7 Hospital Juan A. Fernández, Buenos Aires, Argentina; 8 Centro Integral de Endocrinología y Nutrición (CIEN) Center, Argentina

**Keywords:** Embolization, Hepatectomy, Radiofrequency, Portal vein., Embolização, Hepatectomia, Radiofrequência, Veia porta.

## Abstract

**Background:**

When a major hepatic resection is necessary, sometimes the future liver remnant is not enough to maintain sufficient liver function and patients are more likely to develop liver failure after surgery.

**Aim:**

To test the hypothesis that performing a percutaneous radiofrecuency liver partition plus percutaneous portal vein embolization (PRALPPS) for stage hepatectomy in pigs is feasible.

**Methods:**

Four pigs (Sus scrofa domesticus) both sexes with weights between 25 to 35 kg underwent percutaneous portal vein embolization with coils of the left portal vein. By contrasted CT, the difference between the liver parenchyma corresponding to the embolized zone and the normal one was identified. Immediately, using the fusion of images between ultrasound and CT as a guide, radiofrequency needles were placed percutaneouslyand then ablated until the liver partition was complete. Finally, hepatectomy was completed with a laparoscopic approach.

**Results:**

All animals have survived the procedures, with no reported complications. The successful portal embolization process was confirmed both by portography and CT. In the macroscopic analysis of the pieces, the depth of the ablation was analyzed. The hepatic hilum was respected. On the other hand, the correct position of the embolization material on the left portal vein could be also observed.

**Conclusion:**

“Percutaneous radiofrequency assisted liver partition with portal vein embolization” (PRALLPS) is a feasible procedure.

## RESUMO

**Racional:**

Quando grande ressecção hepática é necessária, às vezes, o fígado remanescente não é suficiente para manter a função hepática e os pacientes são mais propensos a desenvolver insuficiência hepática após a operação.

**Objetivo:**

Testar a hipótese de que a realização de uma divisão do fígado com radiofreqüência percutânea mais a embolização percutânea da veia porta (PROPS) para a hepatectomia regrada em porcos é viável.

**Métodos:**

Quatro porcos (Sus scrofa domesticus) ambos os sexos com pesos entre 25 a 35 kg foram submetidos à embolização percutânea da veia porta com espirais da veia porta esquerda. Por TC contrastada, a diferença entre o parênquima hepático correspondente à zona embolizada e a normal foi identificada. Imediatamente, usando a fusão de imagens entre ultrassom e CT guiada, as agulhas de radiofrequência foram colocadas percutaneamente e depois foram cortando até a partição do fígado estar completa. Finalmente, a hepatectomia foi completada com abordagem laparoscópica.

**Resultados:**

Todos os animais sobreviveram aos procedimentos, sem complicações. O sucesso do processo de embolização do portal foi confirmado por portografia e CT. Na análise macroscópica das peças, analisou-se a profundidade da ablação. O hilo hepático foi respeitado. Por outro lado, a posição correta do material de embolização na veia porta esquerda também pôde ser observada.

**Conclusão:**

“Partição do fígado assistida por radiofrequência percutânea com embolização da veia porta” (PRALLPS) é um procedimento viável.

## INTRODUCTION

When a major hepatic resection is necessary, sometimes the future liver remnant (FLR) is not enough to maintain sufficient liver function and patients are more likely to develop liver failure after surgery^9^ ^,^ ^10^. In order to avoid that undesirable situation, in patientes with normal liver function and with less than 20-30% of FLR, percutaneous portal vein embolization (PVE) used to be the gold standard to achieve its hypertrophy. Although it is a good approach and a technique with a high success rate, it takes between four to six weeks to achieve the goal of hypertrophy, and meanwhile, the tumors could go on growing and even worse, appearing more^8^. To improve that, Schnitzbauer et al^21^ introduced a novel technique called associating liver partition and portal vein ligation for staged hepatectomy (ALPPS). Is a procedure with two steps. The first one consist in an open surgery in which is performed a ligation of the portal branches feeding the side to be resected plus a liver partition. The second step is the hepatectomy. This technique was proven to increase the FLR in less than 10 days and in between 40-80% volume growth by avoiding the formation of collateral vessels^26^. It was a promising approach except for high morbidity and mortality rates which raise to more than 70% and 10% respectively^8^. For that reason, many variants of this technique have been developed^**26**^. Among them, Mini ALLPS was described by De Santibañes et al^6^. Despite of being a less complex procedure, still remains as a two stage open surgery with no despicable morbidity^8^. Then,Jiao et al^16^introduced the splitting of liver parenchyma assisted with radiofrequency performed laparoscopically and named it as radiofrequency assisted liver partition with portal vein ligation (RALPP). Also, other sources of energies have been used in animals^19^ and also in humans such as Gringeri et al^12^ called “laparoscopic microwave ablation and portal vein ligation for staged hepatectomy (LAPS)”. They all have something in common: the less invasive approach in order to reduce morbidity and mortality. 

Therefore, to keep on this evolution, in this study we presenta novel technique called “Percutaneous radiofrequency assisted liver partition with portal vein embolization” (PRALLPS) to demonstrate its feasibility. 

## METHODS

### Animals and protocol

The present study is a prospective and experimental study in animals approved by the Ethics Committee of IHU. It has been held in IHU Strasbourg, France in conjunction with the DAICIM Foundation from December 2016 to July 2017.

The 3 R ethic principles (refinement, replacement and reduction) has been strictly adhered to^6^ ^,^ ^16^. Four pigs (Sus scrofa domesticus)both sexes with weights between 25 to 35 kg were used. The animals were housed in individual cages, respecting the circadian cycle of light-darkness, and with a constant humidity and temperature. The environment was enrichedby the presence of toys. The day before surgery, the experimental subject had been fasted for 24 h, but with free access to water. Anxiety related to moving the cage to the operating room and/or imaging platform was controlled by an intramuscular injection of ketamine (20 mg/kg) + azaperone (2 mg/kg, Stresnil; Janssen-Cilag, Belgium) 1 h before the procedure. Induction was performed with intravenous injection of propofol (3 mg/kg) + pancuronium (0.2 mg/kg). Anesthesia was maintained with 2% isoflurane. Pigs were sacrificed by injection of a lethal dose of general potassium chloride anesthesia.

The study protocol consisted of intervention (PVE plus radiofrequency liver partition), euthanasia in two pigs and liver explantation, and second intervention in the remaining two pigs (laparoscopic hepatectomy) and afterwards euthanasia. 

### Tecnique of PVE and percutaneous radiofrequency liver partition

**FIGURE 1:**
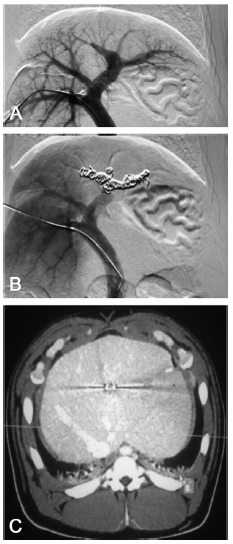
A) Left portal vein showed by portography; B) right portal vein after embolization; C) CT scan with IV contrast after portal vein embolization

The procedure begins with the percutaneous embolization of the left portal vein. For this, an abdominal ultrasound (US) was performed (Acuson S 3000 - Siemens)locating the liver^9^. A branch of the right portal vein was identified. Under US guidance, the vein was accessed using a Chiba 21 G (Cook) needle. The position was confirmed by injecting contrast through the needle under fluoroscopic control (Artis Zeego - Siemens). A portography was done. Once inside the vein, a guide (Guidewire 0.018¨ - Cook) was introduced. The needle was replaced by an introducer (Neff Introducer Set - Cook) using a Seldinger technique. Through the introducer, a catheter (BostonScientific Bern 4 Fr Catheter) was placed in the left branch of the portal vein over accessory guides (Guidewire 0.035¨ Roadrunner - Cook; Guidewire 0.035¨ - Amplatz). The embolizationwas performed using coils of different sizes (Nester Embolization Coils - Cook), including 14x20 mm, 10x20 mm, 8x14 mm, 6x14 mm and 4x14 mm. Correct embolization was confirmed with a final portography^1^. Then, the intrahepatic path was embolized(Veriset Haemostatic Patch - Medtronic, Figures 1 A and B)

Afterwards, a computed tomography (CT, Somatom Definition AS Plus - Siemens) with IV contrast (Ioméron 400 mg/ml - Bracco) was obtained with venous, arterial and portal phases (Figure 1C). A subtle difference was identified between the embolized area and ​​the normal liver parenchyma. 

**FIGURE 2:**
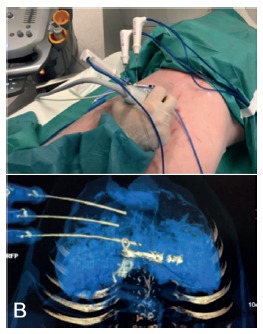
A) Placement the RFA needle using fusion image between US and CT; B) CT scan checking the correct position of the needles

Three simultaneously radiofrequency ablation (RFA) needles (Radiofrequency Cool Trip System Needle - Medtronic) were set in place using fusion of images between US and CT as a guide (Figure 2).They were separated from each other by approximately 2 cm (Radiofrequency Cool Trip Ablation System Equipment - Medtronic). Subsequently, the ablation was performed for 6 min on each needle. The ablation area of each needle was approximately 3 cm in diameter. At the end of each ablation period, the needles were removed and replaced in the same manner by repeating the procedure until complete partitioning along the anterior face of the liver. The border between the parenchyma corresponding to the embolized portal sector and the normal one serves as a reference as well as the right hepatic vein.The depth of the partition was approximately 4.5 cm.

**FIGURE 3:**
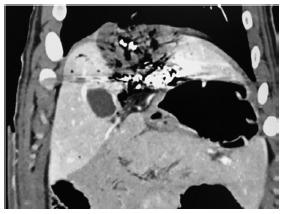
CT scan after ablation

A new CT scan was then repeated, with the same protocol as described above. The liver partition area could be identified, thus confirming the feasibility of the procedure performed so far (Figure 3).

## RESULTS

**FIGURE 4:**
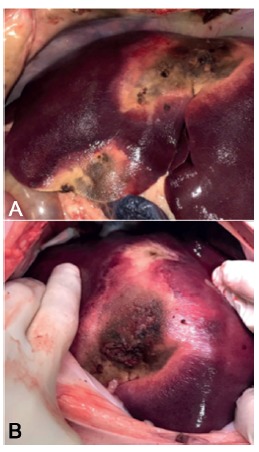
Aspect of liver after ablation: hemihepatectomy by open approach

**FIGURE 5:**
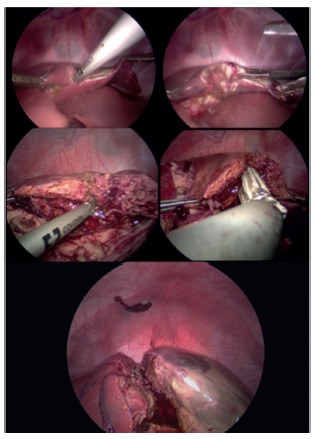
Hemihepatectomy by laparoscopic surgery

The animals were operated on after 2 h of the radiofrequency liver partition.In two pigs, a total hepatectomy was performed after their euthanasia (Figure 4), with the only objective of comparing the CT image of the liver after ablation with the final operative piece. In the remaining two animals, a right hepatectomy was performed by laparoscopy (Karl Storz, Figure 5). The reference for the approach of the liver was the ablation line. Its depth was assesed using translaparoscopy ultrasound (Siemens Acuson P300 LP323 Transducer). To complete the parenchyma partition, we used energy devices (Sonicision Cordless Ultrasonic Dissector 5 mmx39 cm - Covidien) and staplers for the vascular and biliary parts (Stapler Endo GIA - Covidien, Stapler Endo GIA Articulating Reload with Tri-Staple Technology 45 mm Vascular/Medium - Covidien). We used six reloads in one surgery and five in the remainder. Finally, the piece was removed through a medial incision.

All animals survived the procedures. There were no bleeding complications. The two pigs that underwent laparoscopic resection were sacrificed at the end of it. 

There were no complications during the ablation period directly related to this procedure. However, during laparoscopic surgery, small areas of ablation have been observed outside the desired area, such as the spleen of the pig and asmall area in gallbladder (without perforation).

**FIGURE 6:**
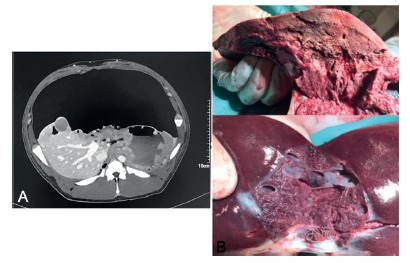
A) CT after laparoscopic hepatectomy; B) right hepatic lobe, right portal vein branch and gallbladder.

It was not necessary to suspend proceedings or take any action. CT with IV contrast after liver laparoscopic resection showed a good vascularized liver remnant (Figure 6A).

In the macroscopic analysis of the pieces, the depth of the ablation was analyzed (Figure 6B).The hepatic hilum was respected. On the other hand, the correct position of the embolization material on the left portal vein could be also observed (Figure 6B).

## DISCUSSION

In most of major liver resections, the percutaneous PVE is the gold standard to achieve the hypertrophy of FLR. Although it has a high success rate, it takes to much time to achieve hypertrophy, and meanwhile, the tumors could increse their size^8^ and if the FLR does not enhanced its volume enough, patients lose precious time. A complementary procedure for the PVE is the embolization of the ipsilateral hepatic vein. This can be done simultaneously with the PVE or sequentially. The former one has the disadvantage of being an expensive procedure with severe potential complications perfomed in many patients that would have been achieve the hipertrophy even without the hepatic vein embolization. The latter, has the same time issue as the PVE alone^18^

In this scenario, the introduction of the ALPPS technique showed to be a major change^8,12,21.^. It has allowed to perform hepatectomies of greater parenchyma volume without presenting postoperative hepatic insufficiency and in much less time than PVE^4^ ^,^ ^7^ ^,^ ^20^. Its disadvantage is being a major surgery in two stages, with a high percentage of associated morbidity and mortality. In order to reduce them, the original technique was modified by the development of the new mini-ALPPS technique^26^ and later also performed by laparoscopy. 

On the other hand, the radiofrequency ablation have shown an impressive progression in terms of equipment allowing to perfom liver partitioning in major liver surgery^5^ ^,^ ^6^ as well as in the laparoscopic approach of ALLPS^11^ ^,^ ^13^.

In this path, it seems that the development of a new procedure that could increase the FLR in a faster manner with a similar morbidiy and mortality than the PVE, would be the highest goal.

In the present study, wasdemonstrated that not only it is possible to perform the liver partition percutaneously but also the laparoscopic liver resection: both together makes PRALPPS technique. This brand new procedure has two potential benefits: it would reduce the time to achieve the FLR hipertrophy because it uses the same concept as the ALPPS technique and also would reduce its morbidity and mortality rates based on the evidence that the percutaneous procedures have less inflamatory response. 

With respect to the limitations of the present study, we should mention that it was held with a small sample size, sufficient to demonstrate its feaseability but not for analize its safety. Regarding this, we experienced two posible complications: the unwanted ablation of the spleen and the gallbladder. It must take into account that most ALPPS procedures are related to right, non-left liver resections as in this study. In addition, the anatomical arrangement of the pig spleen is completely different to the human one. Beyond these special considerations, the correct position of the needle within the hepatic parenchyma when initiating the ablation is very important to avoid these problems^**22**^. Probably, the use of new needles with smaller ablation areas could be a potencial solution in the future. 

## CONCLUSION

Percutaneous radiofrequency assisted liver partition with portal vein embolization (PRALLPS) is a feasible procedure. However, new studies to asses its security should be carried out. 

## References

[1] Angle JF, Siddiqi NH, Wallace MJ Quality Improvement Guidelines forPercutaneous Transcatheter Embolization.

[2] Chan AC, Chok KS, Das JW (2017). Impact of split completeness on future liver remnant hypertrophy in associating liver partition and portal vein ligation for staged hepatectomy (ALPPS) in hepatocellular carcinoma Complete-ALPPS versus partial-ALPPS. Surgery.

[3] Cillo U (2015). Totally Laparoscopic Microwave Ablation and Portal Vein Ligation for Staged Hepatectomy A New Minimally Invasive Two-Stage Hepatectomy. Ann Surg Oncol.

[4] Court FG, Wemyss-Holden SA, Morrison CP (2003). Segmental nature of the porcine liver and its potential as a model for experimental partial hepatectomy. Br J Surg.

[5] Croome KP, Mao SA, Glorioso JM (2015). Characterization of a porcine model for associating liver partition and portal vein ligation for a staged hepatectomy. HPB (Oxford).

[6] De Santibañes E, Alvarez FA, Ardiles V (2016). Inverting the ALPPS paradigm by minimizing first stage impact the Mini-ALPPS technique. Langenbecks Arch Surg.

[7] Edmondson MJ, Pucher PH, Sriskandarajah K (2016). Variations and adaptations of associated liver partition and portal vein ligation for staged hepatectomy (ALPPS) Many routes to the summit. Surgery.

[8] Eshmuminov D, Raptis DA, Linecker M (2016). Meta-analysis of associating liver partition with portal vein ligation and portal vein occlusion for two-stage hepatectomy. Br J Surg.

[9] Mullen JT, Ribero D, Reddy SK (2007). Hepatic insufficiency and mortality in 1,059 noncirrhotic patients undergoing major hepatectomy. J Am Coll Surg.

[10] FERNANDES Eduardo de Souza Martins (2016). THE LARGEST WESTERN EXPERIENCE WITH HEPATOPANCREATODUODENECTOMY: LESSONS LEARNED WITH 35 CASES. ABCD, arq. bras. cir. dig.

[11] Gall TM, Sodergren MH, Frampton AE (2015). Radio-frequency-assisted Liver Partition with Portal vein ligation (RALPP) for liver regeneration. Ann Surg.

[12] Gringeri E, Boetto R, D´Amico FE, Bassi D, Cillio U (2015). Laparoscopic microwave ablation and portal vein ligation for staged hepatectomy (LAPS) a minimally invasive first-step approach. Ann Surg.

[13] Guiu B, Chevallier P, Denys A (2016). Simultaneous trans-hepatic portal and hepatic vein embolization before major hepatectomy the liver venous deprivation technique. Eur Radiol.

[14] Hernández S (2006). El modelo animal en las investigaciones biomédicas. Biomedicina.

[15] Hwang S, Ha TY, Ko GY (2015). Preoperative Sequential Portal and Hepatic Vein Embolization in Patients with Hepatobiliary Malignancy. World J Surg.

[16] Jiao LR, Hakim DN, Gall TM (2016). A totally laparoscopic associating liver partition and portal vein ligation for staged hepatectomy assisted with radiofrequency (radiofrequency assisted liver partition with portal vein ligation) for staged liver resection. Hepatobiliary Surg Nutr.

[17] Moris D, Vernadakis S, Papalampros A (2016). Mechanistic insights of rapid liver regeneration after associating liver partition and portal vein ligation for stage hepatectomy. WorldJGastroenterol.

[18] Munene G, Parker RD, Larrigan J (2013). Sequential preoperative hepatic vein embolization after portal vein embolization for extended left hepatectomy in colorectal liver metastases. World J Surg Oncol.

[19] Ron C (2017). Gaba, James T Bui, Rajyasree Emmadi, et al. Ablative Liver Partition and Portal Vein Embolization: Proof-of-Concept Testing in a Rabbit Model. J Vasc Interv Radiol.

[20] Schadde E, Schnitzbauer AA (2015). Systematic Review and Meta-Analysis of Feasibility, Safety, and Efficacy of a Novel Procedure: Associating Liver Partition and Portal Vein Ligation for Staged Hepatectomy. Ann Surg Oncol.

[21] Schnitzbauer AA, Lang SA, Goessmann H (2012). Right portal vein ligation combined with in situ splitting induces rapid left lateral liver lobe hypertrophy enabling 2-staged extended right hepatic resection in small-for-size settings. Ann Surg.

[22] SURJAN Rodrigo Cañada Trofo, MAKDISSI Fábio Ferrari, MACHADO Marcel Autran Cesar (2015). Anatomical basis for the intrahepatic glissonian approach during hepatectomies. ABCD, arq. bras. cir. dig.

[23] Smith JA, Jennings M (1998). Ethics training for laboratory animal users. Lab Anim.

[24] TORRES Orlando Jorge M, FERNANDES Eduardo S M, HERMAN Paulo (2015). ALPPS: PAST, PRESENT AND FUTURE. ABCD, arq. bras. cir. dig.

[25] Wilms C, Mueller L, Lenk C (2008). Comparative Study of Portal Vein Embolization Versus Portal Vein Ligation for Induction of Hypertrophy of the Future Liver Remnant Using a Mini-Pig Model. AnnSurg.

[26] Zhang GQ, Zhang ZW, Lau WY, Chen XP (2014). Associating liver partition and portal vein ligation for staged hepatectomy (ALPPS) a new strategy to increase resectability in liver surgery. Int J Surg.

